# Association of the Cervical Microbiota With Pregnancy Outcome in a Subfertile Population Undergoing *In Vitro* Fertilization: A Case-Control Study

**DOI:** 10.3389/fcimb.2021.654202

**Published:** 2021-09-23

**Authors:** Xinyao Hao, Pingping Li, Shanshan Wu, Jichun Tan

**Affiliations:** ^1^ Center of Reproductive Medicine, Department of Obstetrics and Gynecology, Shengjing Hospital of China Medical University, Shenyang, China; ^2^ Key Laboratory of Reproductive Dysfunction Disease and Fertility Remodeling of Liaoning Province, Shenyang, China

**Keywords:** 16S r RNA, IVF (*in vitro* fertilization), pregnancy, infertility, cervical microbiota

## Abstract

The microorganisms of the reproductive tract have been implicated to affect *in vitro* fertilization (IVF) outcomes. However, studies on the reproductive tract microbiota of infertile women are limited and the correlation between cervical microbiota and IVF outcome remains elusive. This study aimed to characterize the cervical microbiota of IVF patients undergoing embryo transfer (ET) and assess associations between the cervical microbiota and pregnancy outcomes while exploring the underlying contributing factors. We launched a nested case-control study of 100 patients with two fresh or frozen-thawed cleavage embryos transferred per IVF cycle. Cervical swabs were collected on the day of ET and divided into four groups according to clinical pregnancy outcomes. Variable regions 3 and 4 (V3-V4) of the 16S rRNA gene were amplified and sequenced on the Illumina MiSeq platform. In fresh IVF-ET cycles, the clinical pregnancy group (FP, n = 25) demonstrated higher α diversity (*P* = 0.0078) than the non-pregnancy group (FN, n = 26). Analysis of similarity (ANOSIM) revealed a significant difference in β diversity between the two groups (R = 0.242, *P* = 0.001). In frozen-thawed ET cycles, though not significant, similar higher α diversity was found in the clinical pregnancy group (TP, n = 27) compared to the non-pregnancy group (TN, n = 22) and ANOSIM analysis showed a significant difference between the two groups (R = 0.062, *P* = 0.045). For patients in fresh IVF-ET groups, *Lactobacillus*, *Akkermansia*, *Desulfovibrio*, *Atopobium*, and *Gardnerella* showed differentially abundance between pregnant and non-pregnant women and they accounted for the largest share of all taxa investigated. Among them, *Lactobacillus* was negatively correlated with the other genera and positively correlated with serum estradiol levels. Logistic regression analysis suggested that the composition of the cervical microbiota on the day of ET was associated with the clinical pregnancy in fresh IVF-ET cycles (*P* = 0.030). Our results indicate that cervical microbiota composition has an impact on the outcome of assisted reproductive therapy.

## Introduction

At least a billion microorganisms settle on the female reproductive tract and interact with the host to maintain a series of physiological processes such as immunity and metabolism (Jie et al., 2021). Disruption of human microbial stability is the leading cause of infections and is also implicated in other diseases such as Crohn’s disease, Subglottic stenosis, and periodontitis (Peñalver Bernabé et al., 2018; Curtis et al., 2020; Aronson et al., 2021; Siniagina et al., 2021). Cervicovaginal microbiota is known to play an important role in female reproductive function. Healthy cervicovaginal microbiota is often characterized by a low diversity of bacterial species, with *Lactobacillus* tending to be the dominant microbiota. In recent years, researchers divided the cervicovaginal microbiota of women at childbearing age into six main community state types (CSTs), of which four were predominated by either *Lactobacillus crispatus* (CST I), *Lactobacillus gasseri* (CST II), *Lactobacillus iners* (CST III) or *Lactobacillus jensenii* (CST V), and two (CST IV-A and CST IV-B) comprised a wide array of strict and facultative bacterial anaerobes, where CST IV-A was characterized with the higher abundance of BVAB1 (Elovitz et al., 2019). The composition of the vaginal microbiota is affected by various factors including race, personal hygiene, sexual activity, and menstrual cycle (Gajer et al., 2012), with a shift to facultative or strictly anaerobic bacterial dominance causing the clinical syndrome called bacterial vaginosis (BV) (Mendling, 2016). Previous studies have demonstrated the association between BV and adverse obstetric outcomes such as late-term abortion and premature delivery (Nelson et al., 2007; Foxman et al., 2014).

Infertility refers to a couple’s failure to become pregnant after one year of regular and unprotected intercourse (Cooper et al., 2009), affecting up to 10% of couples at childbearing age worldwide (Mascarenhas et al., 2012). Since the first live birth achieved by *in vitro* fertilization (IVF) in 1978, improving the pregnancy rate of IVF patients has become a major clinical challenge (De Geyter, 2019). Research has shown that—in addition to the known factors used in prediction models such as female age, sperm quality, and antral follicle count—IVF outcome might also be affected by the microorganisms of the female reproductive tract (Koedooder et al., 2019). The relatively few studies on the microbiota inhabited reproductive tract of infertile women have yielded inconsistent results on the correlation between vaginal microbiota and IVF outcome. Liversedge et al evaluated the vaginal swabs collected at the time of oocyte retrieval by Gram stain and found that the incidence of BV in tubal infertility was significantly higher than that in non-tubal infertility and that BV did not affect fertilization (Liversedge et al., 1999). Ralph et al. showed that BV was associated with an increased risk of miscarriage in the first trimester of women undergoing IVF (Ralph et al., 1999). In another study, researchers used Nugent score and polymerase chain reaction to diagnose BV in IVF patients and found no significant difference in obstetric results between the BV group and the non-BV group (Mangot-Bertrand et al., 2013). Results of the study conducted by Selim et al. showed that BV and lower concentrations of hydrogen peroxide-producing *Lactobacillus* may reduce the conception rate and increase the rate of failed pregnancy on women who were undergoing intracytoplasmic sperm injection (Selim et al., 2011). A recent meta-analysis conducted on IVF patients showed that BV was significantly associated with early spontaneous abortion, but had no significant effect on live birth rate and clinical pregnancy rate (Haahr et al., 2019).

With the development of high-throughput sequencing technology, emerged data have identified the existence of continuous changed microbiota along the female reproductive tract. In 2016, Moreno et al. demonstrated the presence of endometrial microbiota and found that its composition was associated with the reproductive results of IVF patients (Moreno et al., 2016). Chen et al. systematically sampled the microbiota in the reproductive tract of 110 women at childbearing age and performed 16S rRNA gene sequencing. The results revealed that the vaginal-uterine microbiota was a continuum and the microbiota in the cervical canal and uterus was different from the vaginal microbiota (Chen et al., 2017). The cervix, located at the transition zone between the lower and upper reproductive tract, serves as both a mechanical and chemical barrier to ascending bacteria. The state of the uterus is an important maternal factor which affects female fertility, but knowledge about endometrial microbiota was deficient owing to the invasiveness of uterine sample collection. Cervical microbiota detected from sampling of cervical mucosa, can be used to survey the status of the uterus and peritoneal cavity in the general population with minimally invasive procedures (Chen et al., 2017). Based on the special anatomy of the cervix, the risk of contamination at the sampling point is minimal (Schoenmakers and Laven, 2020). Our study aimed to characterize the cervical microbiota of 100 women undergoing IVF treatment and assess the impact of cervical microbiota composition on IVF clinical pregnancy outcomes.

## Materials and Methods

### Patient Recruitment

This study recruited infertile female patients undergoing IVF treatment at the Reproductive Center of Shengjing Hospital of China Medical University from January 2019 to March 2019. The study was approved by the Ethics Committee of the Shengjing Hospital of China Medical University and all participants provided written informed consent (approval number: 2017PS269K). Inclusion criteria were as follows: 20–40 years of age; undergoing assisted reproductive technology (ART) treatment with their own gametes; transfer of two cleavage-stage embryos. Exclusion criteria were as follows: autoimmune diseases; endocrine diseases; cervical diseases; endometrial diseases (uterine fibroids, adenomyosis, moderate to severe endometriosis, unrecoverable uterine adhesion, etc.); blood contamination of collected samples. All participants followed the conventional ART protocol for treatment and the primary outcome of clinical pregnancy was defined as positive fetal heartbeat and fetal buds observed under ultrasound 35 days after embryo transfer (ET). The baseline characteristics collected for each patient included age, body mass index (BMI), duration of infertility, smoking history, drinking history, menstrual cycle length, cause of infertility, previous fertility history, the total dose of Gonadotropin (Gn), duration of Gn administration, number of oocytes retrieved, endometrial thickness on the day of ET, estradiol (E_2_) and progesterone (P) levels, and number of good-quality embryos transferred.

### Sample Collection

In the operating room, two cervical samples were collected before ET: 1) a sterile cotton ball was used to clean the patient’s vaginal secretions; 2) two sterile cotton swabs were used to access the patient’s cervical canal and rotated to obtain cervical secretions. Both swabs were used for genomic DNA extraction. During this process, operator ensured that the cotton swab did not touch the patient’s vaginal wall. After collecting the swab samples, ultrasound-guided ET was performed according to the established protocol. All samples were stored at –80°C for later analysis (Chen et al., 2017).

### DNA Extraction and PCR Amplification

Microbial DNA was extracted from cotton swab samples using the PureLink microbiota DNA extraction Kit (ThermoFisher) according to the manufacturer’s protocol. The V3-V4 region of the bacteria 16S ribosomal RNA genes was amplified by PCR (95°C for 3 min, followed by 30 cycles at 98°C for 20 s, 58°C for 15 s, and 72°C for 20 s and a final extension at 72°C for 5 min) using primers 341F 5’-CCTACGGGRSGCAGCAG-3’ and 806R 5’-GGACTACVVGGGTATCTAATC-3’. PCR reactions were performed in 30 μL mixture containing 15 μL of 2 × KAPA Library Amplification ReadyMix, 1 μL of each primer (10 μM), 50 ng of template DNA, and ddH_2_O.

### Illumina MiSeq PE250 Sequencing

Amplicons were extracted from 2% agarose gels and purified using the AxyPrep DNA Gel Extraction Kit (Axygen Biosciences, Union City, CA, U.S.) according to the manufacturer’s instruction and quantified using Qubit^®^2.0 (Invitrogen, U.S.). After preparation of the library, these tags were sequenced on the MiSeq platform (Illumina, Inc., CA, USA) for paired end reads of 250bp, which were overlapped on their 3’ ends for concatenation into original longer tags. DNA extraction, library construction, and sequencing were conducted at Realbio Genomics Institute (Shanghai, China).

### Process of Sequencing Data

Pandaseq (version 2.8.1) was used for reads assemble and Realbio analysis platform (Shanghai, China) was responsible for quality control. Tags, trimmed of barcodes and primers, were further checked on their rest lengths and average base quality. 16S tags were restricted between 220 bp and 500 bp such that the average Phred score of bases was no worse than 20 (Q20) and no more than 3 ambiguous N. The copy number of tags was enumerated and redundancy of repeated tags was removed. Only the tags with a frequency of more than 1, which tend to be more reliable, were clustered into Operational Taxonomic Units (OTUs), each of which had a representative tag. OTUs were clustered with 97% similarity using UPARSE (http://drive5.com/uparse/) and chimeric sequences were identified and removed using Userach (version 7.0). Each representative tags was assigned to a taxa by RDP Classifer (version 2.12, http://rdp.cme.msu.edu/) against the RDP database (version 11.4, http://rdp.cme.msu.edu/) using a confidence threshold of 0.8. Analysis of α diversity (Chao1 index, Shannon index, Simpson index) and β diversity (unweighted UniFrac) was also achieved by python scripts of Qiime (version 1.9.1).

### Statistical Analysis

Statistical analysis was conducted with R software version 3.5.1 and SPSS version 25.

We generated the OTU Venn diagram (in R) to illustrate the number of OTUs shared among four groups or unique to a group, according to the abundance of OTU in each sample. Shannon index and Simpson index were used to evaluate α diversity and P values were calculated using the Wilcox test function in R between the pregnancy group and non-pregnancy group. Analysis of similarity (ANOSIM) and Principal co-ordinates analysis (PCoA) was performed to compare the overall cervical microbiota composition between pregnancy and non-pregnancy groups in fresh and frozen-thawed cycles. Linear discriminant analysis effect size (LEfSe) analysis were performed with the LEfSe tool (http://huttenhower.sph.harvard.edu/galaxy). The cladogram was generated using the online LEfSe project. For the LEfSe analysis, we used the Wilcoxon test to detect significantly different abundances between the pregnancy and non-pregnancy groups in fresh cycles and performed Linear discriminant analysis (LDA) scores to estimate the effect size (threshold: ≥ 2) at all levels. Based on the LEfSe result, we selected the genera abundance with Top 30 and conducted the Spearman correlation heatmap between dominant genera through the corrplot package of R software to show the relationships among dominant genera. The correlation between differential abundances at genus level and serum sex hormone levels is calculated by the Spearman correlation test, and the thermal map is drawn by R software corrplot package so as to reveal the important relationship between differential abundant genera and sex hormone (E_2_, P). In SPSS, Kruskal-Wallis H test, chi-square test, and One way ANOVA were used to compare the baseline data between groups. Univariate and multivariate logistic regression were used to evaluate the association of clinical factors and microbiota composition with clinical pregnancy. The continuous variables with normal distribution were expressed as the mean ± standard deviation (SD), and the variables with non-normal distribution were presented as the median (interquartile range). *P*-value of less than 0.05 is considered significant.

### Accession Number

The sequence data in this study have been deposited in NCBI under BioProject number PRJNA693672.

## Results

### Characteristics of the Participants

A total of 124 patients contributed cervical samples. Among them, the samples from 20 patients were contaminated with blood during the collection process, barring them from further analysis. Samples derived from four patients failed sequencing due to low DNA content. Ultimately, 100 patients were included in the study. A total of 51 patients underwent fresh IVF-ET cycle, 25 of whom achieved clinical pregnancy (Group FP, FP01-FP25) and 26 were non-pregnant (Group FN, FN01-FN26). A total of 49 patients underwent a frozen-thawed ET cycle, 27 of whom were clinically pregnant (Group TP, TP01-TP27) and 22 were non-pregnant (Group TN, TN01-TN22). **Table 1** summarizes the baseline characteristics of these four groups.

**Table 1:** Characteristics of the participants.

		Group FN (n = 26)	Group TN (n = 22)	Group FP (n = 25)	Group TP (n = 27)	*P*-value
Age (years)		33.0 ± 3.9	32.5 ± 4.0	31.2 ± 4.0	31.1 ± 4.4	0.247^a^
BMI (kg/m^2^)		23.2 ± 3.2	23.2 ± 4.0	23.3 ± 4.1	22.8 ± 3.3	0.974^a^
Infertility duration (years)		3.4 ± 2.5	4.3 ± 2.3	3.4 ± 2.1	4.6 ± 2.9	0.238^b^
Smoking	Yes	0	2 (9.1%)	1 (4.0%)	2 (7.4%)	0.524^c^
	No	26 (100%)	20 (90.9%)	24 (96.0%)	25 (92.6%)	
Alcoholism	Yes	0	1 (4.5%)	0	0	0.220^c^
	No	26 (100%)	21 (95.5%)	25 (100%)	27 (100%)	
Menstrual cycle	regular	25 (96.2%)	18 (81.8%)	21 (84.0%)	25 (92.6%)	0.297^c^
	irregular	1 (3.8%)	4 (18.2%)	4 (16.0%)	2 (7.4%)	
Indication^d^	Male factors	10 (38.5%)	9 (40.9%)	13 (52.0%)	10 (37.0%)	0.704^c^
	Tubal factors	14 (53.8%)	16 (72.7%)	13 (52.0%)	19 (70.4%)	0.305^c^
	PCOS	4 (15.4%)	3 (13.6%)	3 (12.0%)	5 (18.5%)	0.962^c^
	Ovarian dysfunction	2 (7.7%)	2 (9.1%)	2 (8.0%)	2 (7.4%)	1.000^c^
	Unexplained	4 (15.4%)	1 (4.5%)	2 (8.0%)	2 (7.4%)	0.657^c^
	Others	1 (3.8%)	1 (4.5%)	4 (16.0%)	2 (7.4%)	0.459^c^
Previous pregnancy times		0.88	0.59	0.56	0.74	0.862^b^
Previous childbirth times		0.15	0.05	0.08	0	0.332^b^
Previous miscarriage times		0.42	0.50	0.36	0.59	0.884^b^
Previous ectopic pregnancy times		0.23	0.00	0.12	0.15	0.166^b^
Previous IVF cycle		0.42	0.50	0.24	0.81	0.082^b^

Values are given as mean ± SD, number (%), and mean. BMI, Body Mass Index; PCOS, polycystic ovary syndrome; IVF, In Vitro Fertilization; FN, fresh IVF-ET cycle non-pregnancy; FP, fresh IVF-ET cycle pregnancy; TN, frozen-thaw ET cycle non-pregnancy; TP, frozen-thaw ET cycle pregnancy.

a By One way ANOVA.

b By Kruskal-Wallis H test.

c By chi-square test.

d Not 100% in total due to multiple diagnoses for some patients.

*P < 0.05.

### Analysis of Cervical Microbiota Profiles

**Figure 1:**
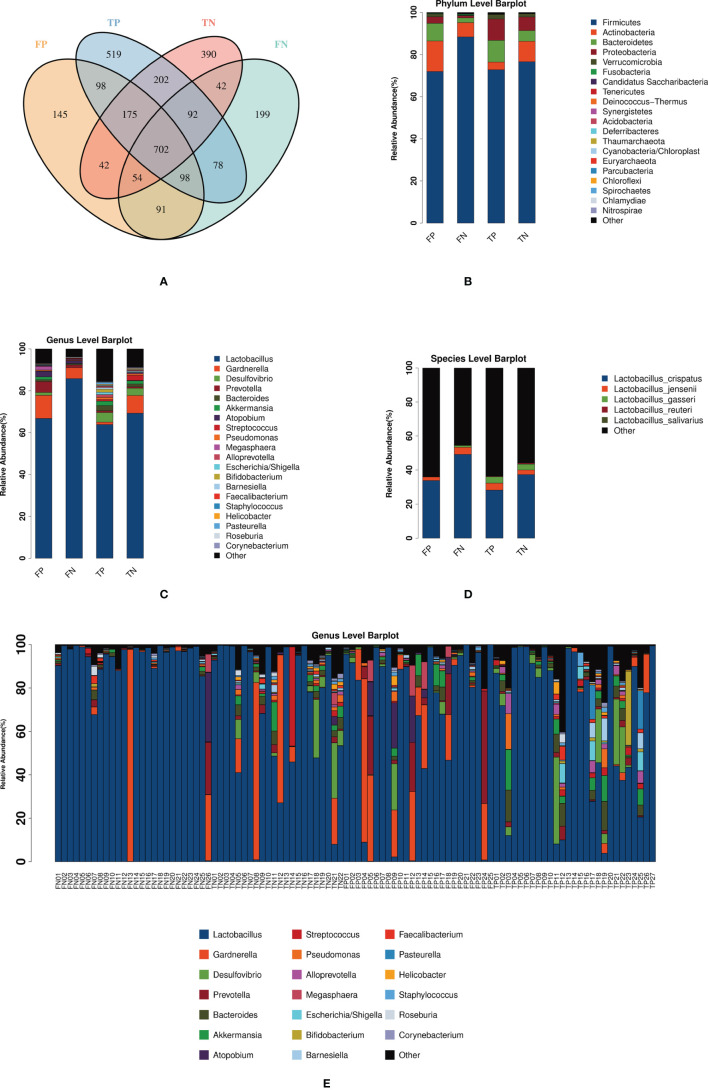
Shows the distribution of the microbiota in the FP, FN, TP, and TN groups. **(A)** Venn diagram: represents the OTU distribution of the four groups. **(B–D)**  The average relative abundance of the sample microbiota in four groups at the level of phylum, genus, and species. **(E)** The relative abundance of the 100 sample microbiota at the genus level. FN, fresh IVF-ET cycle non-pregnancy; FP, fresh IVF-ET cycle pregnancy; TN, frozen-thaw ET cycle non-pregnancy; TP, frozen-thaw ET cycle pregnancy.

A total of 5 704 398 clean reads were generated by 16S rRNA gene sequencing. After using USEARCH to cluster and filter on a similarity of 0.97, 28 122 OTUs were obtained. **Figure 1A** shows the number and distribution of OTUs among the four groups. At the phylum level, Firmicutes predominated in infertile women, followed by Actinobacteria and Bacteroidetes (**Figure 1B**). At the genus level, the cervical microbiota of infertile women consisted primarily of *Lactobacillus*, followed by *Gardnerella*, *Desulfovibrio*, *Prevotella*, and *Bacteroides* (**Figure 1C**). It is worth noting that the average relative abundance of *Lactobacillus* in most cases of FP group (66.76%), FN group (85.82%), TP group (63.84%), and TN group (69.27%) was higher than 60%, with the difference between fresh goups was significant while not significant between frozen-thawed goups (**Table S1**). *Lactobacillus*, as the dominant bacterial genus, consisted of different classifications at the species level. **Figure 1D** showed the average relative abundance of microbiota in four groups at the level of species. Due to technical limitations, only some species have been detected. Among the five types of *Lactobacillus* species detected, *L. crispatus* has the highest average relative abundance. In fresh IVF-ET cycles, the relative abundances of *L. crispatus*, *L. jensenii*, and *L. gasseri* in the pregnancy group were all lower than those in the non-pregnancy group, but these differences were not statistically significant (**Table S1**). Similarly, in the frozen-thawed ET cycles, the relative abundance of *L. crispatus* in the pregnancy group was lower than that in the non-pregnancy group. While the relative abundances of *L. jensenii* and *L. gasseri* in the pregnancy group were higher than those in the non-pregnancy group, these differences again were not statistically significant (**Table S1**). The bar graph in **Figure 1E** shows the cervical microbiota distribution of 100 infertile women at the genus level. Among them, 84% of the samples presented *Lactobacillus* as the dominant bacteria (84/100); 48.8% of these samples were from clinically pregnant women (41/84). Of the remaining 16 samples dominated by other bacteria, 68.8% of patients were clinically pregnant (11/16). The abundance of *Gardnerella* in the cervical microbiota of four patients was greater than 60% (FP04, FN13, TN08, TN12).

**Figure 2:**
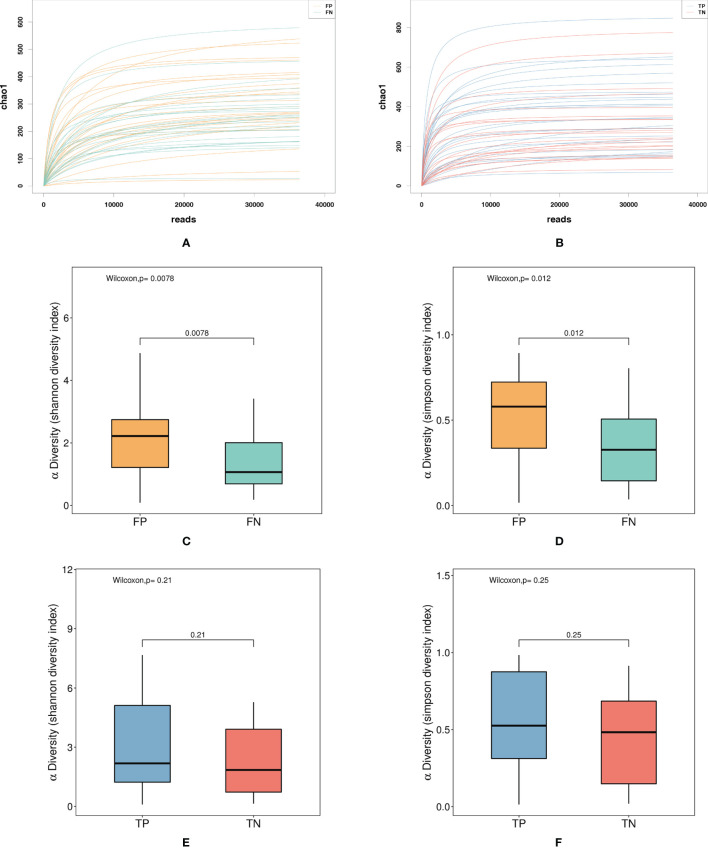
Shows the α diversity of the FP, FN, TP, and TN groups. **(A)** Dilution curve: FP, FN group Chao diversity index dilution curve **(B)** Dilution curve: TP, TN group Chao diversity index dilution curve **(C)** Box diagram, FP, FN group Shannon diversity index **(D)** Box plot: FP, FN group Simpson diversity index **(E)** Box plot: TP, TN group Shannon diversity index **(F)** Box plot: TP, TN group Simpson diversity index. FN, fresh IVF-ET cycle non-pregnancy; FP, fresh IVF-ET cycle pregnancy; TN, frozen-thaw ET cycle non-pregnancy; TP, frozen-thaw ET cycle pregnancy.

The microbiota diversity of the samples from fresh IVF-ET cycles was significantly lower than that of the samples from frozen-thawed ET cycles. The α diversity index dilution curves Chao1 richness (**Figures 2A, B**) of the species abundance of both fresh and frozen-thawed ET cycles showed a smooth trend, indicating that the sequencing depth was sufficient to cover most of the microorganisms in each sample. Shannon and Simpson indices were used to evaluate α diversity. In fresh and frozen-thawed cycles, the α diversity of the pregnancy group was higher than that of the non-pregnancy group; the difference was statistically significant between fresh groups. In fresh IVF-ET cycles, the Shannon index of the non-pregnancy group (mean = 1.345) was significantly lower than that of the pregnancy group (mean = 2.085), with *P* = 0.0078 (**Figure 2C**). The Simpson index of the non-pregnancy group (mean = 0.354) was significantly lower than that of the pregnancy group (mean = 0.537), with *P* = 0.0117 (**Figure 2D**). In the frozen-thawed ET cycle, the Shannon index (mean = 2.221) of the non-pregnancy group was lower than that of the pregnancy group (mean = 2.966), with *P* = 0.2111 (**Figure 2E**). The Simpson index of the non-pregnancy group (mean = 0.455) was lower than that of the pregnancy group (mean = 0.556), with *P* = 0.2503 (**Figure 2F**).

**Figure 3:**
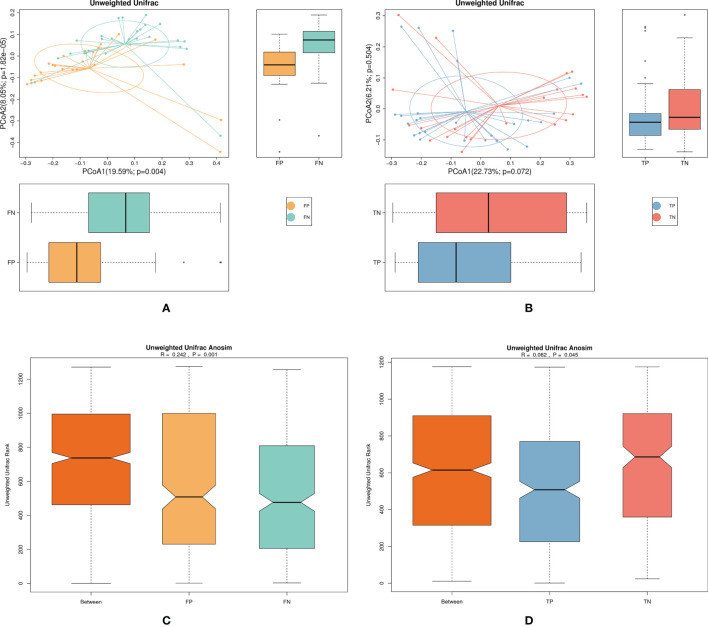
Shows the β diversity of the microbiota in the pregnancy and non-pregnancy groups of the fresh and frozen-thawed cycles. **(A)** FP, FN group microbiota unweighted PCoA analysis chart **(B)** TP, TN group microbiota unweighted PCoA analysis chart **(C)** FP, FN group microbiota unweighted Anosim analysis chart **(D)** TP, TN group microbiota unweighted Anosim analysis chart. FN, fresh IVF-ET cycle non-pregnancy; FP, fresh IVF-ET cycle pregnancy; TN, frozen-thaw ET cycle non-pregnancy; TP, frozen-thaw ET cycle pregnancy, PCoA, Principal co-ordinates analysis; ANOSIM, Analysis of similarity.

We used PCoA and ANOSIM analysis to elucidate the β diversity of the microbiota jointly between pregnancy and non-pregnancy groups in fresh and frozen-thawed cycles. The β diversity analysis aimed to compare the overall microbiota composition between pregnancy and non-pregnancy groups. In fresh IVF-ET cycles, PCoA of the pregnancy and non-pregnancy group samples showed different distributions in the first and second principal coordinates (**Figure 3A**, *P* = 0.004), while in frozen-thawed ET cycles, PCoA revealed similar distributions between the pregnancy and non-pregnancy group samples (**Figure 3B**, *P* = 0.072). In the ANOSIM of both fresh and frozen-thawed ET cycles, R > 0 and *P* < 0.05 revealed significant differences between the pregnancy and the non-pregnancy groups (fresh IVF-ET cycle, R = 0.242, *P* = 0.001; frozen-thawed ET cycle, R = 0.062, *P* = 0.045); the difference between the fresh groups was greater than the difference between the frozen-thawed groups. In addition, R = 0.062 indicated that there was little difference between the pregnancy and non-pregnancy group samples in the frozen-thawed cycles (**Figures 3C, D**).

**Figure 4:**
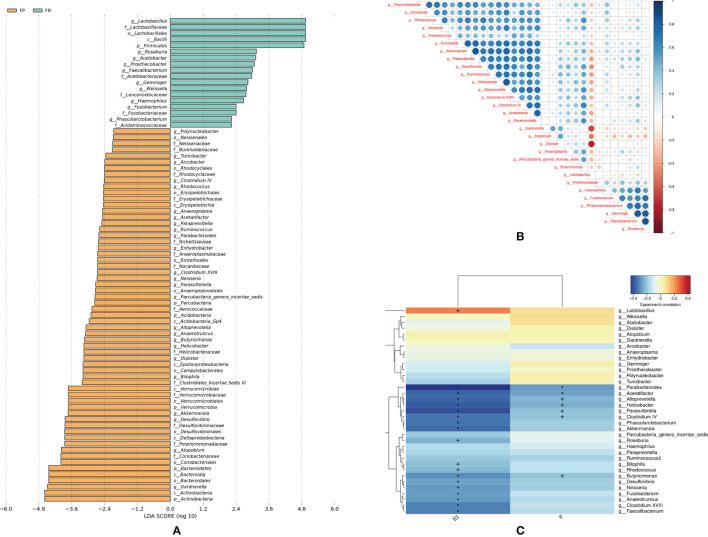
Shows the differential abundance and association analysis among the cervical microbiota of the fresh pregnancy group and the non-pregnancy group. **(A)** LEfSe analysis chart: The LDA score of the genera which showed differentially abundance between pregnant and non-pregnant women in fresh IVF-ET cycle. **(B)** Spearman correlation coefficient analysis: the important patterns and relationships among the genera abundance with Top 30 in FP, FN groups **(C)** Spearman thermal map analysis of the correlation between the serum sex hormone levels and genera which were detected in LEfSe analysis. FN, fresh IVF-ET cycle non-pregnancy; FP, fresh IVF-ET cycle pregnancy. E_2_, estradiol; LDA, Linear discriminant analysis; LEfSe, Linear discriminant analysis effect size analysis; P, progesterone. ^+^ *P* < 0.05; *P < 0.01.

Given that the α diversity and β diversity in the fresh IVF-ET cycle groups were statistically different, we further conducted LEfSe analyses, in which LDA score was used to estimate the effect of the abundance of each component on a different effect. As shown in **Figure 4A**, the clustering result of bacterial taxa with different abundance at different levels can be observed. At the genus level, abundance of 35 genera was different between pregnancy and non-pregnancy groups (LDA scores more than 2.0, **Table S2**) in the fresh IVF-ET cycle. Among these genera, the LDA scores of *Lactobacillus*, *Akkermansia*, *Desulfovibrio*, *Atopobium*, and *Gardnerella* were greater than 3.6, indicating a relatively large degree of difference between the groups. Furthermore, Spearman correlation coefficient analysis of the top 30 genera exhibiting differential abundance demonstrated that *Lactobacillus* had a negative correlation with other genera, of which the strongest negative correlation was with *Gardnerella* and *Dialister* (**Figure 4B**). To identify the underlying contributing factors for the relative abundance of the 35 genera which were detected based on LEfSe analysis, we assessed the serum sex hormone levels (E_2_, P) on the day of ET and conducted a Spearman thermal map analysis of the correlation between the serum sex hormone levels and genera abundance. The results showed that the abundance of these genera had a strong correlation with the serum sex hormone levels on the day of transplantation. As shown in **Figure 4C**, the abundance of 19 genera was correlated with the E_2_ level (*Lactobacillus*, *Parabacteroides*, *Acetatifactor*, *Alloprevotella*, *Helicobacter*, *Parasutterella*, *Clostridium IV*, *Phascolarctobacterium*, *Akkermansia*, *Roseburia*, *Bilophila*, *Rhodococcus*, *Butyricimonas*, *Desulfovibrio*, *Neisseria*, *Fusobacterium*, *Anaerotruncus*, *Clostridium XVIII*, *Faecalibacterium*). Among them, the abundance of *Lactobacillus* was positively correlated with E_2_, while the remaining genera were negatively correlated with E_2_. The abundance of seven genera was negatively correlated with the P level (*Parabacteroides*, *Parasutterella*, *Acetatifactor*, *Alloprevotella*, *Helicobacter*, *Clostridium IV*, *Butyricimonas*).

### Multivariate Analysis

In fresh IVF-ET cycles, the diversity between the microbiota of the pregnancy and non-pregnancy groups was statistically different; we ultimately selected five genera, (*Lactobacillus*, *Gardnerella*, *Atopobium*, *Akkermansia*, *Desulfovibrio*) with large differences between the groups based on the results of LEfSe analysis for further analysis. We defined Log (*Lactobacillus*/others) as the logarithmic conversion of the relative abundance ratio of *Lactobacillus* to the other four genera. In view of the fact that in addition to microbiota, embryonic and maternal factors may also have impacts on clinical pregnancy, we compared the endometrial thickness, E_2_, embryo quality, and other related clinical factors between the pregnancy and non-pregnancy group (**Table 2**). Cleavage embryos graded above 8CII on day 3 were considered as good-quality embryos (Wu et al., 2020). Moreover, we conducted univariate logistic regression including age, BMI, endometrial thickness, and other factors that may affect the clinical pregnancy rate as independent variables. As shown in **Table 3**, Log (*Lactobacillus*/others) and the number of good-quality embryos transferred were correlated with clinical pregnancy. Consequently, we included these two variates in the multivariate logistic regression analysis (**Table 4**). The result revealed that the number of good-quality embryos transferred was associated with increased odds of clinical pregnancy and a lower ratio of *Lactobacillus* to other bacteria in the cervical microbiota was associated with decreased odds of clinical pregnancy, while only the latter was statistically significant.

**Table 2:** Supplement of baseline data for fresh IVF-ET cycle patients.

	Non-pregnancy (n = 26)	Pregnancy (n = 25)	*P*-value
E_2_ (pg/mL)	1333 (689.75, 1778.75)	1548 (1111, 2352.5)	0.407
Total Gn dosage (IU)	2400 (1762.5, 3017)	2475 (1837.5, 3000)	0.741
Gn days (days)	10 (8, 11.25)	10 (8.5, 11.5)	0.901
Endometrial thickness (mm)	10 (8.75, 12)	10 (10, 12)	0.518
Number of follicles	8.5 (6, 15.25)	11 (8, 16)	0.160
Number of good-quality embryo transferred	2 (1, 2)	2 (2, 2)	0.024^*^

Values are given as median (25^th^, 75^th^ percentile).

Tested by Kruskal-Wallis H test. ^*^P < 0.05.

E_2_, estradiol; Gn, Gonadotropin.

**Table 3:** Univariate logistic regression assessing the association of clinical factors and microbiota composition with clinical pregnancy.

	B	OR	95% Cl	*P*-value
**Age (years)**	-0.121	0.886	0.762-1.030	0.114
**BMI (kg/m2)**	0.011	1.011	0.868-1.077	0.893
**Infertility duration**	0.011	1.011	0.795-1.284	0.931
**E_2_ (pg/mL)**	0.000	1.000	1.000-1.001	0.652
**Total Gn dosage (IU)**	0.000	1.000	0.999-1.001	0.714
**Gn days (days)**	0.000	1.000	0.777-1.286	0.998
**Endometrial thickness (mm)**	0.041	1.042	0.856-1.269	0.681
**Number of follicles**	0.074	1.077	0.975-1.188	0.143
**Log (*Lactobacillus*/others)**	-0.515	0.597	0.394-0.906	0.015*
**Number of good-quality embryo transferred**	1.269	3.557	1.178-10.738	0.024*

BMI, Body Mass Index; E_2_, estradiol; Gn, Gonadotropin; OR, odds radio; 95% CI, 95% confidence interval. ^*^P < 0.05.

**Table 4:** Multivariate logistic regression assessing the association of the number of good-quality embryos transferred and microbiota composition with clinical pregnancy.

	B	OR	95% Cl	*P*-value
**Log (*Lactobacillus*/others)**	-4.57	0.633	0.419-0.957	0.030*
**Number of good-quality embryo transferred**	1.174	3.234	0.984-10.634	0.053

OR, odds radio; 95% CI, 95% confidence interval. ^*^P < 0.05.

## Discussion

Over the past decade, several researchers have assessed the reproductive tract microbiota of female patients undergoing IVF through 16S rRNA sequencing technology (Hyman et al., 2012; Moreno et al., 2016; Bernabeu et al., 2019). To the best of our knowledge, this is the first study on the cervical microbiota of IVF patients with different clinical pregnancy outcomes. Our results suggested that, whether in fresh or frozen-thawed ET cycles, *Lactobacillus* is the predominant genus present in the cervical microbiota of IVF patients. In fresh IVF-ET cycles, the cervical microbiota in the pregnancy and non-pregnancy groups showed differences in α diversity and β diversity. Moreover, *Lactobacillus*, *Akkermansia*, *Desulfovibrio*, *Atopobium*, and *Gardnerella* were differentially abundant between pregnant and non-pregnant women and they had the LDA scores of all taxa investigated. Among them, *Lactobacillus* was negatively correlated with other genera and positively correlated with serum E_2_ levels. Ultimately, we found that both the composition of the cervical microbiota and the number of good-quality embryos transferred were significantly correlated with the clinical pregnancy rate in fresh IVF-ET cycles.

Our results revealed that the α diversity of the pregnancy group was higher than that of the non-pregnancy group, regardless of the cycle and the difference was statistically significant in fresh IVF-ET cycles. This result is inconsistent with the differences between endometrial and vaginal microbiota reported in previous studies (Moreno et al., 2016; Bernabeu et al., 2019). We consider the main reason for the differences in the conclusions of these studies to be related to the different sites for microbiota collection. Our understanding of the female reproductive tract microbiota is gradually changing. Previously, researchers speculated that vaginal bacteria colonized the upper genital tract through the cervix, thereby affecting pregnancy outcomes (Mitchell et al., 2015). In a 2016 study, the researchers conducted two samplings of vaginal and endometrial microbiota on women at childbearing age during the same menstrual cycle. The results showed that the vaginal microbiota was different from the endometrial microbiota, also indicating that the endometrial microbiota is not completely derived from the vagina (Moreno et al., 2016). In 2017, Chen et al. sampled and sequenced the reproductive-tract microbiota of 110 women at childbearing age. They found that the community types of some subjects were different in the cervix and endometrium; moreover, the microbiota from the vagina to the peritoneal fluid was continuous changing (Chen et al., 2017). The results of a study in 2020 validated the previous conclusion. The researchers collected samples from the lower third of the vagina, posterior fornix, cervical mucus, endometrium, and peritoneal fluid of patients with endometriosis for sequencing. The results showed that the cervical mucus of endometriosis patients began to show significant differences in community diversity that increased upward the reproductive tract (Wei et al., 2020). These continuous changes in the female reproductive tract microbiota indicate that research on the cervical microbiota must be more in-depth to provide a greater understanding of its potential impact on pregnancy outcomes.

*Lactobacillus* was still the predominant genus in the cervical microbiota of most IVF patients, which is consistent with the previous study (García-Velasco et al., 2017). *Lactobacillus* dominates the lower genital tract of women at reproductive age mainly *via* utilizing the glycogen deposited under the action of estrogen (Spear et al., 2014; Nasioudis et al., 2015) and impeding the growth of other bacteria through metabolized lactic acid (O’Hanlon et al., 2013; Roselló et al., 2013), competitive inhibition (Boris and Barbés, 2000) or bacteriocin and other substances production (Ojala et al., 2014). Of interest, we found that the relative abundance of *Lactobacillus* dominated patients in the non-pregnancy group was significantly higher than the clinical pregnancy group of fresh cycles, while non-significant difference was seen between frozen-thawed groups. In consistence, Bernabeu et al. reported that the abundance of vaginal *Lactobacillus* on the day of ET in women who achieved pregnancy was not significantly different from the control group after frozen embryo transfer (Bernabeu et al., 2019). It could be found that the E_2_ level on the day of embryo transfer was higher in the fresh groups than in the frozen-thawed groups (**Table S3**). E_2_ concentrations have been reported to decrease from the day of human chorionic gonadotropin (hCG) trigger to ET and this decrease was significantly slight in patients who did not have a live birth (Hyman et al., 2012). Fluctuations during ovulation and especially low levels during the periovulatory period of vaginal *Lactobacillu*s in women was also observed (Zhao et al., 2020). These findings indicate that the high abundance of Lac*tobacillus* in *the* non-pregnancy group of fresh cycles may be partially due to the maintained high level of E_2_ though its concentrations on the day of hCG were not recorded. Thus future longitudinal research that collecting more comprehensive information is necessary to clarify this phenomenon.

Moreover, compared with the results of Vergaro’s research on vaginal bacterial communities (76.7% *Lactobacillus* dominance) (Vergaro et al., 2019), our 16S rRNA results showed a lower percentage of *Lactobacillus* dominance of the cervical microbiota (73%). Nor did we uncover a positive correlation between *L. crispatus* and clinical pregnancy outcome (**Table S4**). It has been reported by Chen et al. that distinct microbial communities exist in a continuum along the female reproductive tract, involving the vagina, cervical canal, fallopian tubes, uterus, and peritoneal fluid (Chen et al., 2017). The specific anatomy of the cervix and cervical mucus may function as a partial ascent filter (Mitchell et al., 2015), exerting an impact on the composition of the cervical microbiota. Regarding *L. crispatus—*validated by Koedooder et al. to be a predictor of IVF outcome—a favorable profile with < 60% *L. crispatus* dominance of the vaginal microbiota indicated the highest chance of pregnancy among the other grouping strategies (Koedooder et al., 2019). The results of this study suggest that *L. crispatus* may have a more complex relationship with the clinical pregnancy rate.

Beyond the predominance of *Lactobacillus*, non-*Lactobacillus*-dominanced samples were also detected in our pregnancy groups. Despite the fact that numerous preceding studies reported that communities lacking *Lactobacillus* as the dominant strain were not conducive to pregnancy outcomes (Moreno et al., 2016; Wee et al., 2018; Liu et al., 2019; Singer et al., 2019), non-*Lactobacillus*-dominanced microbiota was seen in healthy or pregnant people with less exceptional. Reid suggested that the presence of non-*Lactobacillus* organisms does not necessarily confer disease (Reid, 2016). A trial in 2019 also revealed that some patients achieved ongoing pregnancies with 0% *Lactobacillus* in the endometrium (Hashimoto and Kyono, 2019). This team failed to prove the obvious benefits of establishing an endometrium dominated by *Lactobacillus* in pregnancy outcomes (Kyono et al., 2019). These conflicting experimental results suggest that we must redefine the community most beneficial to IVF outcomes. When the cervical microbiota presents non-*Lactobacillus*-dominance, clinical pregnancy can still be achieved.

Age, duration of infertility, and other factors also have an impact on the clinical pregnancy rate (Brosens et al., 2004; Eijkemans et al., 2008). We ultimately used multivariate logistic regression analysis to analyze the impact of various factors on the clinical pregnancy rate, deviating from previous research (Moreno et al., 2016; Bernabeu et al., 2019). Based on the results of univariate logistic regression analysis, we selected two indicators—the ratio of *Lactobacillus* to other bacteria in the cervical microbiota and the number of good-quality embryos transferred—as independent variables. Finally, through multivariate regression analysis, both the ratio of *Lactobacillus* to other bacteria in the cervical microbiota and the quality of the embryos transferred contributed to the IVF clinical pregnancy rate.

Unfortunately, due to the small sample size used in our research, we cannot validate our model. In addition, our research did not involve the collection of specimens at other time points and other locations in the reproductive tract, rendering some of our assertions unverifiable. By sequencing variable regions (V3-V4) of the 16S rRNA gene, the ability to characterize members of the cervical microbial community to species-level taxonomy was limited. These constitute the limitations of our experiment.

In conclusion, the results of this study indicate that the cervical microbiota has an impact on the outcome of IVF. Although the relative abundance of *Lactobacillus* may be related to the clinical pregnancy rate, it is impossible to conclude that non-*Lactobacillus*-based microorganisms are not conducive to pregnancy. Further studies must clarify the optimal abundance of *Lactobacillus* in patients undergoing IVF treatment and explore the mechanisms by which multiple microbiotas affect the IVF outcomes.

## Data Availability Statement

The original contributions presented in the study are publicly available. This data can be found here: NCBI, PRJNA693672.

## Ethics Statement

The studies involving human participants were reviewed and approved by the Ethics Committee of the Shengjing Hospital of China Medical University. The patients/participants provided their written informed consent to participate in this study.

## Author Contributions

JT and XH designed and were responsible for this project. JT and XH collected samples and conducted clinical studies. XH and SW performed statistical analysis on the data. XH and PL wrote this paper. XH and PL revised the manuscript. All authors contributed to the article and approved the submitted version.

## Funding

This work was supported by the National Key Research and Development Program of China (2018YFC1004203), Central Government Special Fund for Local Science and Technology Development (2020JH6/10500006), and Shengjing Freelance Researcher Plan of Shengjing Hospital of China Medical University.

## Conflict of Interest

The authors declare that the research was conducted in the absence of any commercial or financial relationships that could be construed as a potential conflict of interest.

## Publisher’s Note

All claims expressed in this article are solely those of the authors and do not necessarily represent those of their affiliated organizations, or those of the publisher, the editors and the reviewers. Any product that may be evaluated in this article, or claim that may be made by its manufacturer, is not guaranteed or endorsed by the publisher.
