# Supplementary material for: Association of the Cervical Microbiota With Pregnancy Outcome in a Subfertile Population Undergoing In Vitro Fertilization: A Case-Control Study
Source: Front Cell Infect Microbiol. 2021 Sep 23;11:654202. doi: 10.3389/fcimb.2021.654202 (PMC8495128; doi:10.3389/fcimb.2021.654202)
Supplement: Supplementary file 1 [file Table_1.docx]

Supplementary Table 1.

The relative abundance of the *Lactobacillus* and *Lactobacillus* species in the four groups.

|  | FP | FN | *P_FP vs.FN_* | TP | TN | *P_TP vs.TN_* |
| --- | --- | --- | --- | --- | --- | --- |
| *Lactobacillus* | 66.77 ± 36.36 | 85.83 ± 26.32 | **0.038*** | 63.84 ± 35.39 | 69.28 ± 32.76 | 0.583 |
| *L. crispatus* | 33.81 ± 34.78 | 49.18 ± 36.96 | 0.133 | 28.20 ± 35.71 | 37.33 ± 39.77 | 0.402 |
| *L. jensenii* | 2.13 ± 5.36 | 4.16 ± 8.24 | 0.303 | 4.12 ± 19.05 | 2.69 ±7.51 | 0.742 |
| *L. gasseri* | 0.12 ± 0.27 | 1.07 ± 3.75 | 0.208 | 3.76 ± 11.92 | 3.44 ± 10.82 | 0.922 |
| *L. reuteri* | 0.17 ± 0.24 | 0.16 ± 0.25 | 0.816 | 0.33 ± 0.59 | 0.73 ± 2.75 | 0.464 |

Data was presented as mean ± SD. Tested by Student’s t-test or Mann-Whitney U test. **P* < 0.05

FN: fresh IVF-ET cycle non-pregnant; FP: fresh IVF-ET cycle pregnant; TN: frozen-thaw ET cycle non-pregnant; TP: frozen-thaw ET cycle pregnant

Supplementary Table 2.

The LDA scores and *P* value of 35 genera differentially abundant between pregnancy and non-pregnancy groups in fresh IVF-ET cycle.

| genus | enrich group | LDA score | *P*-value |
| --- | --- | --- | --- |
| *Lactobacillus* | FN | 4.945797 | 0.024947 |
| *Roseburia* | FN | 3.149721 | 0.000072 |
| *Acetobacter* | FN | 3.119891 | 0.022483 |
| *Prosthecobacter* | FN | 3.067155 | 0.023486 |
| *Faecalibacterium* | FN | 3.004824 | 0.008333 |
| *Gemmiger* | FN | 2.835561 | 0.013552 |
| *Weissella* | FN | 2.794133 | 0.040642 |
| *Haemophilus* | FN | 2.674172 | 0.038957 |
| *Fusobacterium* | FN | 2.409976 | 0.01121 |
| *Phascolarctobacterium* | FN | 2.241443 | 0.01066 |
| *Polynucleobacter* | FP | 2.062212 | 0.025969 |
| *Turicibacter* | FP | 2.352992 | 0.003504 |
| *Arcobacter* | FP | 2.390922 | 0.007524 |
| *Clostridium IV* | FP | 2.411171 | 0.026058 |
| *Rhodococcus* | FP | 2.429447 | 0.002504 |
| *Anaeroplasma* | FP | 2.470474 | 0.037726 |
| *Acetatifactor* | FP | 2.476314 | 0.000057 |
| *Paraprevotella* | FP | 2.499969 | 0.003259 |
| *Ruminococcus* | FP | 2.578715 | 0.00073 |
| *Parabacteroides* | FP | 2.604873 | 0.040194 |
| *Enhydrobacter* | FP | 2.629185 | 0.046541 |
| *Clostridium XVIII* | FP | 2.661859 | 0.012698 |
| *Neisseria* | FP | 2.66874 | 0.010008 |
| *Parasutterella* | FP | 2.721621 | 0.024523 |
| *Parcubacteria_genera_incertae_sedis* | FP | 2.746388 | 0.035597 |
| *Alloprevotella* | FP | 3.075644 | 0.001285 |
| *Anaerotruncus* | FP | 3.088683 | 0.035597 |
| *Butyricimonas* | FP | 3.130395 | 0.001751 |
| *Helicobacter* | FP | 3.152781 | 0.001639 |
| *Dialister* | FP | 3.155938 | 0.013319 |
| *Bilophila* | FP | 3.185137 | 0.025589 |
| *Akkermansia* | FP | 3.726977 | 0.017285 |
| *Desulfovibrio* | FP | 3.840952 | 0.000285 |
| *Atopobium* | FP | 3.976838 | 0.028053 |
| *Gardnerella* | FP | 4.480048 | 0.048905 |

Tested by Linear discriminant analysis Effect Size analyse.

LDA: Linear discriminant analysis; FN: fresh IVF-ET cycle non-pregnant; FP: fresh IVF-ET cycle pregnant.

Supplementary Table 3.

The serum estradiol level on the embryo transfer day of four groups.

|  | FP | FN | TP | TN | *P*-value |
| --- | --- | --- | --- | --- | --- |
| E_2_ (pg/mL) | 1548(1111, 2352.5) | 1333(689.75,1778.75) | 186(110, 274)^a,b^ | 238(145, 628)^a,b^ | 0.000^*^ |

Values are given as median (25^th^ , 75^th^ percentile).

Tested by Kruskal-Wallis *H* test. ^*^ *P* < 0.05

E_2_: estradiol

^a^ *P* < 0.05 compared with the FP group (*P_FP vs.TP_* = 0.000, *P_FP vs.TN_* = 0.000)

^b^ *P* < 0.05 compared with the FN group (*P_FN vs.TP_* = 0.000, *P_FN vs.TN_* = 0.004)

Supplementary Table 4.

Univariate logistic regression assessing the association of the *Lactobacillus* species with clinical pregnancy.

|  | B | OR | 95%Cl | *P*-value |
| --- | --- | --- | --- | --- |
| *L. crispatus* | -0.012 | 0.988 | 0.972 - 1.004 | 0.132 |
| *L. jensenii* | -.047 | 0.954 | 0.871 - 1.046 | 0.314 |
| *L. gasseri* | -0.917 | 0.400 | 0.079 - 2.029 | 0.268 |
| *L. reuteri* | 0.279 | 1.322 | 0.132 - 13.209 | 0.812 |

OR: odds radio; 95% CI: 95% confidence interval. * *P* < 0.05
